# Strain-Level Characterization of *Legionella* Environmental Isolates via MALDI-TOF-MS

**DOI:** 10.3390/microorganisms11010008

**Published:** 2022-12-20

**Authors:** David Otto Schwake, Todd Sandrin, Lin Zhang, Morteza Abbaszadegan

**Affiliations:** 1 Department of Natural Sciences, Middle Georgia State University, 100 University Pkwy, Macon, GA 31206, USA; 2 School of Mathematical and Natural Sciences, New College of Interdisciplinary Arts & Sciences, Arizona State University at the West Campus, 4701 W. Thunderbird Road, Glendale, AZ 85306, USA; 3 China Innovation Center, Shimadzu (China) Co., Beijing Branch, Beijing 100020, China; 4 School of Sustainable Engineering and the Built Environment, Arizona State University at the Tempe Campus, 1151 S. Forest Ave, Tempe, AZ 85281, USA

**Keywords:** MALDI, *pneumophila*, typing, cluster analysis, water distribution systems

## Abstract

As a waterborne pathogen of increasing concern, techniques for cost-effective and rapid characterization of *Legionella* are vital. This study examines the development of a Matrix Assisted Laser Desorption/Ionization-Time of Flight Mass Spectrometry (MALDI-TOF-MS) analysis methodology for this microbe. First, optimal sample preparation methods for the analysis of environmental *Legionella* isolates via MALDI-TOF-MS were determined. These methods were then implemented to perform strain-level characterization of environmental *Legionella* isolates from central Arizona. Results demonstrate that a MALDI-TOF-MS method involving BCYE agar-based culturing and protein extraction-based sample preparation yield high-quality mass spectra. Twenty-eight environmental *Legionella* isolates originating from two separate drinking water distribution systems were analyzed. Multiple species were detected, and strain-level characterization was achieved, with 12 unique strains distinguished. In addition, isolates of *L. pneumophila*, the most common species observed in the study, were correctly assigned to specific sampling sites. These results demonstrate the potential for this technique to be applied for sub-species characterization of *Legionella* with significant benefits over established methodologies.

## 1. Introduction

Since the discovery of Legionnaires’ disease in 1976, bacteria of the genus *Legionella*, particularly *L. pneumophila*, have become water-borne pathogens of significant concern, causing more drinking-water related disease outbreaks than any other pathogen in the United States [1]. Continually rising legionellosis incidence, as well as the variety of sources of transmission [2,3], highlight the need for tools to aid in the rapid identification and characterization of these pathogens for public health purposes, such as tracking transmission sources during outbreak investigations. Due to the frequency with which these organisms are found in public water supplies and the low exposure event-to-disease rate, advances in high-resolution, rapid strain typing methodology are particularly relevant for *Legionella*.

While immunological and genetic approaches have been successfully used for the typing of *Legionella* [4,5], these methods can be considered time consuming and costly, two characteristics which are significantly improved upon through the use of Matrix Assisted Laser Desorption Time of Flight-Mass Spectrometry (MALDI-TOF-MS) [6]). MALDI-TOF-MS has been used increasingly in recent years as a tool for clinical microbiologists to efficiently identify pathogens. Recently, this technology has been proposed as an alternative to conventional methods for the typing of microbes and studies have shown promising results for a number of organisms, particularly those of clinical relevance. *Escherichia coli* isolates have been successfully identified based on pathotype [7], and MALDI-TOF-MS was found to be a more rapid alternative to repetitive sequence based-PCR for the typing of drug resistant *Acinetobacter* [8]). In addition to clinical microbes, this technology has been successfully applied to the typing of environmental isolates of a number of bacteria including lactic acid bacteria, *E. coli*, and *Enterococcus* spp. [9,10,11,12].

While work has been performed on the use of MALDI-TOF-MS for the identification of *Legionella* species in clinical settings, fewer studies have examined *Legionella* isolated from environmental samples. Gaia [4] compared MALDI-TOF MS data from hundreds of clinical, water, cooling tower, and soil isolates of various *Legionella* species to conventional sequence-based identification. Moliner [13] demonstrated a high level of sensitivity and specificity for the use of MALDI-TOF-MS in the identification of clinical and environmental *Legionella*. Svarrer and Uldum [14] examined the distribution of *Legionella* species in clinical and environmental isolates from Denmark using MALDI-TOF-MS. Pennanec [15] investigated the refinement of a MALDI-TOF-MS protocol for the identification of environmental bacteria, applying it successfully to *Legionella* obtained from a cooling tower. While these and other studies have advanced research on the application of MALDI-TOF-MS for *Legionella* species and genus-level identification, little work has reported strain-level differentiation of *Legionella* [16] using this promising technology. By validating their results via comparison to pulsed-field gel electrophoresis-based typing, they demonstrated the capability for this method to distinguish strains between *L. pneumophila* isolates from two isolated hot springs in Japan. Two recent studies have furthered this area of research, examining *L. pneumophila* isolates sourced from environmental or clinical samples. Identification of serotypes [17] and sequence types [3] of these isolates via MALDI-TOF MS serves to highlight the advancement of techniques for strain-level identification of *Legionella* and other environmental pathogens. The successful methodology of these and other studies demonstrate that the ability to reliably type *Legionella* strains from various environmental water samples via MALDI-TOF-MS would have tremendous applicability to the medical, public health, and water industries [18].

As an environmental pathogen of increasing concern, techniques for cost-effective and rapid characterization of *Legionella* are vital, thus demonstrating the relevancy of research aimed at improving the MALDI-TOF-MS analysis methodology for these bacteria. The goals of this study were twofold: (1) to identify optimal preparation methods for the analysis of environmental *Legionella* isolates via MALDI-TOF-MS, and (2) to implement these methods to perform strain-level typing on environmental *Legionella* isolates from central Arizona, USA. By conducting a systematic and quantitative analysis of methodology optimization for the analysis of *Legionella* via MALDI-TOF-MS, followed by the utilization of this methodology to type several strains of this microbe originating from separate and varied environmental sampling sites, this study directly demonstrates the potential for the application of this technology regarding this pathogen.

## 2. Materials and Methods

### 2.1. Environmental Sampling

Environmental water samples were collected from sources originating from two drinking water distribution systems in central Arizona, USA. Sampling site selection was based on the following criteria: drinking water systems in central Arizona, systems with different water sources, and systems separated geographically. One system, located west of Phoenix, AZ, consisted of chlorinated ground water, while the other system, located east of Phoenix, AZ, consisted of conventionally treated chlorinated surface water. The west system contained water with properties unusual for drinking water, including low/sporadic treatment, high salinity, fecal contamination, and high levels of *Legionella* contamination [19]. A total of 12 *Legionella* isolates from the west system were analyzed: 3 from tap water and 9 from automobile washer fluid reservoirs filled with washer fluid prepared using the system’s tap water. A total of 11 *Legionella* isolates from the east system were analyzed, all from tap water. All but two samples derived from the east system (samples E5 and E6) were sourced from a model drinking water distribution system previously inoculated with a laboratory strain of *L. pneumophila* Knoxville-1 ATCC strain 33153 (American Type Culture Collection, Manassas, VA, USA). A stock culture of Knoxville-1 33153 was also analyzed, alongside 4 isolates of Knoxville-1 33153 sampled from laboratory experiments in sterile water. A total of 28 isolates were analyzed in this study: one Knoxville-1 stock culture isolate (K), 4 Knoxville-1 isolates from laboratory experiments(L1–4), 11 east system environmental isolates (E1–10), and 12 west system environmental isolates (W1–11) (Table 1).

### 2.2. Legionella Culturing

All water samples were collected and processed and all *Legionella* isolates were cultivated using previously described methods [20]. BD BBL Buffered Charcoal Yeast Agar (Diagnostic Systems, Sparks, MD, USA) media was used for *Legionella* culturing, with glycine, polymixin B, vancomycin, and cycloheximide supplemented for environmental water samples. Water samples with low concentrations of *Legionella* were concentrated via membrane filtration using 0.45 micron cellulose filters (Millipore Corp., Bedford, MA, USA). Environmental water samples with high levels of background growth were subjected to heat treatment at 50 °C for 30 min prior to plating [21]. All samples were cultured at 37 °C for 72 h, with an additional 96 h if necessary for full colony formation. Colonies presumed to be *Legionella* based on morphology were simultaneously cultured onto BCYE and Tryptic Soy Agar (Diagnostic Systems, Sparks, MD, USA) for confirmation. Culture confirmed *Legionella* isolates were stored at −80 °C in a long-term storage solution comprised of 75% Charcoal Yeast Extract Broth (CYE), 15% Glycerol, and 10% Millipore water (Millipore Corp., Bedford, MA, USA). One liter of CYE media contained the following: activated carbon (2.0 g), yeast extract (10.0 g), ferric pyrophosphate (0.25 g), L-cysteine HCL (0.4 g), and distilled water (1000 mL).

### 2.3. DNA Extraction and Molecular Analysis

A PCR method [21] was performed to determine if isolates belonged to the *Legionella* genus and *L. pneumophila* species. These tests were also used to confirm Knoxville-1 33153 isolates. DNA extraction was performed on isolated colonies derived from environmental samples using a ZYMO Research yeast/bacterial DNA extraction kit (Zymo Research Corporation, Irvine, CA, USA). *L. pneumophila* specific *mip* gene primers LpneuF (5′-CCGATGCCACATCATTAGC-3′) and LpneuR (5′-CCAATTGAGCGCCACTCATAG-3′), as well as *Legionella* genus 16S rRNA primers LEG-225 (5′-AAGATTAGCCTGCGTCCGAT-3′) and Leg-858 (5-GTCAACTTATCGCGTTTGCT-3′) were used. The PCR amplification mixture used consisted of 12.5 µL Promega GoTaq Green MasterMix (Promega Biosciences LLC., San Luis Obispo, CA, USA), 10 µL DNA template, and 0.13 µM each primer, with a final reaction volume of 25 µL. Gel electrophoresis was performed in a 1% agarose gel containing 0.05 µL/mL of 10,000X Invitrogen SYBR Safe DNA Gel Stain (Life Technologies Corporation, Carlsbad, CA, USA) to detect PCR products.

### 2.4. MALDI-TOF-MS Sample Preparation

Isolates analyzed via MALDI-TOF-MS were prepared either from broth or agar cultures. For broth cultures, isolates were plated from storage onto BCYE and incubated for 72–120 h. Isolated colonies were then inoculated into 5 mL of CYE and incubated with shaking at 150 RPM for 72–120 h. Cell density was normalized for all cultures to an optical density at 600 nm of 3 ± 0.2 OD units. The cultures were then centrifuged at 15,000× *g* for 5 min, with the resulting supernatant decanted. After resuspending in 1 mL of Millipore water, a second centrifugation at 15,000× *g* for 5 min was performed, followed by resuspension in Millipore water and a third centrifugation at 15,000× *g* for 5 min. Supernatant was removed and the resulting pellets were subjected to further preparation. For agar cultures, isolates were plated from storage onto BCYE and incubated for 72–120 h. Colonies were then removed from the plates and suspended in 1 mL of sterile Millipore water. Cell density was normalized to an optical density at 600 nm of 3 ± 0.2 OD units. The cultures were then centrifuged at 15,000× *g* for 5 min, with the resulting supernatant decanted. The resulting supernatants were then removed and the resulting pellets were subjected to further preparation.

Two sample preparation methods were used for MALDI TOF-MS analysis: intact cell (IC) and protein extraction (PE) preparations. For IC preparations, pellets were resuspended in 100 µL of Millipore water by vortexing for 30 s. 100 µL of alpha-Cyano-4-hydrocinnamic acid matrix solution consisting of 50% acetonitrile (Sigma Chemical Company, Bedford, MA, USA), 2.5% trifluoroacetic acid (Alfa Aeser, Ward Hill, MA, USA), and 47.5% Millipore water, with alpha-Cyano-4-hydrocinnamic acid added to saturation were added to the suspension followed by an additional 30 s of vortexing. An amount of 2 µL of the supernatant from this preparation were spotted to a 96-well ground steel target plate (Bruker Daltonics, Bilerica, MA, USA) and allowed to air dry. For PE preparations, pellets were suspended in 25 µL of 70% formic acid (Avantor Performance Materials, Center Valley, PA, USA), and vortexed for 30 s. Acetonitrile (25 µL) was then added to the suspension, followed by an additional 30 s of vortexing. The suspensions were then centrifuged at 15,000× *g* for 5 min. A quantity of 1 µL of the resulting supernatants were then spotted to a 96-well polished steel target plate and allowed to air dry. An amount of 1 µL of an alpha-Cyano-4-hydrocinnamic acid matrix solution was then overlayed onto each sample and allowed to air dry. Data from this study came from isolates cultured and prepared on three separate days. All preparations analyzed were spotted in triplicate. Both preparation methods effectively inactivate *Legionella* cultures as determined by previously detailed cultivation techniques.

### 2.5. MALDI-TOF-MS Data Acquisition and Analysis

All samples were analyzed using a Bruker Microflex LRF MALDI-TOF Mass Spectrometer (Bruker Daltonics, Billerica, MA, USA) with a nitrogen laser (λ = 337 nm) operating in positive linear mode. Calibration was performed prior to data collection using the following mass calibrants (Sigma Aldrich, St. Loius, MO, USA): ACTH 1–17 (2094 Da), ACTH 18–36 (2466 Da), insulin oxidized B (3494 Da), insulin (5734 Da), Cytochrome C (12,360 Da), and Myoglobin (16,952 Da) suspended in matrix solution at a ratio of 1:1. FlexControl 3.0 software (Bruker Daltonics, Billerica, MA, USA) was used to operate the instrument. Automatic data acquisition was performed for the samples using the following settings: laser power 25–75%, 2000–20,000 Da range, peak evaluation with resolution higher than 100, random walk with 10 shots at raster spot, and 300 satisfactory shots summed up in 100 shot steps.

Raw spectrum data were converted to text files using FlexAnalysis 3.0 (Bruker Daltonics, Billerica, MA, USA) before preprocessing and further analyses, which were performed in BioNumerics 7.1 (Applied Maths, Austin, TX, USA). Spectra were preprocessed in BioNumerics 7.1 using the software’s default settings for relaxed peak detection. Baseline subtraction was performed using a rolling disc method, smoothing via a Kaiser Window filter, and peak detection via a continuous wavelet transform ridge algorithm with a signal-to-noise ratio of 5.

### 2.6. MALDI-TOF-MS Optimization

To determine the optimum sample preparation and cell culturing methods for the environmental isolates used in the study, MALDI-TOF-MS data obtained from IC and PE sample preparations (with agar culturing) were compared for spectra quality, followed by samples prepared from agar and broth cultures (with PE sample preparation). For the agar/broth culture comparison, four environmental isolates were analyzed: two from the east system and two from the west system were compared alongside two stock cultures of Knoxville-1 33153. For the IC/PE comparison, 13 environmental isolates were analyzed, 7 from the east system (E2/4/5/7/9/10/11), and 6 from the west system (W5/6/7/8/11/12), alongside two stock cultures of Knoxville-1 33153. Identical data acquisition and analysis methods were applied to both sets of comparisons. The following parameters for peak quality were then determined for samples from the comparisons: base peak signal to noise ratio, base peak resolution, and peak range. Average values, along with their standard deviations, were calculated for triplicate samples of each culture condition or sample preparation method. Similarity matrices derived from cluster analysis were used to determine technical replicate reproducibility.

### 2.7. MALDI-TOF-MS Cluster Analysis

Cluster analysis was performed on spectra acquired from 34 cultures of the 28 isolates analyzed. All sample preparations used in this cluster analysis were prepared via agar/PEMS methods determined to be optimum conditions for spectra quality. After preprocessing of raw spectra in Bionumerics 7.1, triplicate technical replicates of each isolate were summarized using the software’s summary spectra function to generate one composite mass spectrum (with a high level of stringency) for that isolate. Note that a single replicate from isolate L1 produced a spectrum with fewer than 5 peaks, suggesting low-quality analysis likely due to user error, and it was removed from the cluster analysis. For L1, only two technical replicates were summarized to generate the composite spectrum. Cluster analysis was performed using a complete linkage cluster analysis algorithm based on the Pearson correlation similarity coefficient. To determine the species level similarity percentage cut-off value in the cluster analysis performed, similarities between known *L. pneumophila* isolates (previously determined via PCR) were compared. Based on this rationale, a 32% similarity value was chosen. A similar approach was taken to determine the strain level cut-off value of 90% similarity by comparing similarities of biological replicates of four isolates (K, W2, W5, and W10). A methodology for strain characterization similar to this has been performed previously [22], in which strains of the fungus *Trichophyton rubrum* were distributed into sub-groups based on similarity values of 85% derived via cluster analysis of MALDI-TOF MS spectra.

### 2.8. 16S rRNA Analysis of Isolates

Multiple sequence alignment performed in Clustal Omega [23] was used to compare 16S rRNA sequences generated via Sanger sequencing of PCR products (primers previously described) from several isolates. Isolates were chosen based on putative strain and species assignment from cluster analysis of MALDI-TOF-MS spectra to examine isolates from multiple predicted strains. The isolates analyzed were: ATCC 33153 *L. pneumophila* (K), two non-*pneumophila* isolates (E11 and W11), three east sampling site *L. pneumophila* isolates (E2, E4, and E5), and four west valley isolates (W5, W6, W7, and W8).

### 2.9. Statistical Analysis

To determine whether the effects of the various preparation methods tested had a significant effect on the spectrum quality metrics examined, Student’s T-tests were performed, with a *p*-value cut-off value of 0.05 used to determine significance using R 3.0.2 (R Foundation for Statistical Computing, Vienna, Austria). Jackknife analysis using average similarity was performed in Bionumerics 7.1 to quantify the ability of cluster analysis performed to correctly group isolates.

## 3. Results and Discussion

### 3.1. MALDI-TOF-MS Optimization

To evaluate sample culturing and preparation methods ideal for examining environmental isolates of *Legionella* via MALDI-TOF-MS, Agar/IC and Agar/PE preparation, as well as Broth/IC and Broth/PE were compared. Analyses of spectra quality for IC and PE preparations for three isolates, K, W5, and W11, were chosen for display due to their representation of the range of results observed in the study (Table 2a). W5 had the most similar spectra quality using the two methods, while W11 had the most differing. Table 2b displays the results of broth culturing with PE preparation for the same three strains.

Base peak signal to noise ratios were higher in PE preparation for all isolates except for IC preparation for W5. Base peak resolution was higher in PE preparation for all isolates, although not significantly so for W5. Peak numbers were greater in IC preparations (substantially higher in broth culture) for all isolates. Reproducibility was higher for PE preparation for all isolates, although not significantly so for W5 (Table 2a). It should be noted that certain isolates regularly produced spectra of unacceptably low quality using broth versus agar culturing and IC versus PE preparation, occasionally to the point where data acquisition was impossible. For example, when prepared via broth culturing and PE preparation, only one of three replicate platings of W11 produced spectra of high enough quality for analysis (Table 2b).

Overall data of metrics for MALDI-TOF-MS spectra quality of *Legionella* comparing the two preparation and culturing methods suggest that agar culturing and PE preparation were ideal for the techniques performed. While other comparisons have shown no significant difference in preparation quality between differing methods [24], consistently higher reproducibility was observed from this combination of preparation approached on isolates examined in this study. This improved quality of results when applying these two methods for the *Legionella* isolates demonstrates their potential for use in high resolution MALDI-TOF-MS analysis. The reason for the higher reproducibility values seen from these methods may be attributed to the higher base peak signal to noise ratios, lower base peak mass variations, and greater base peak resolutions observed between triplicate samples of most isolates tested. These metrics of spectrum quality reflect the ability to distinguish background from sample, precisely measure peak mass, and distinguish unique peaks [25], all of which are of great importance for MALDI-TOF-MS analysis broadly but become increasingly necessary as the target level of taxonomic resolution increases [26]. An additional benefit of PE preparation compared to smear or IC approaches is the likely increased biosafety as whole cells (inactivated or not) are placed on MALDI target plates with this method. With the potential to aerosolize target material, respiratory pathogens such as *Legionella* that frequently enter a viable but non-culturable state are less likely to pose a safety risk if intact cells are not analyzed. Refining MALDI-TOF-MS preparation and analysis methods to increase these metrics will most likely play a key role in the advancement of bacterial strain-level characterization via this technology.

Peak number, a metric commonly used to determine spectra quality, appeared to be negatively correlated to reproducibility for the majority of analyzed isolates. This may be attributed to the lower signal to noise ratios observed: analysis of samples prepared via IC preparation and broth culturing was less efficient at distinguishing background from significant peaks, resulting in larger amounts of peaks. The relationship between reproducibility of this metric may indicate that its usefulness in measuring MALDI-TOF-MS spectra quality does not apply to all organisms or preparation methods and could warrant further study. Interestingly, while agar culturing produced significantly higher quality spectra for all isolates tested than broth culturing, the disparities in quality metrics observed between IC and PE preparations varied between isolates. This is seen in the reproducibility values obtained (Table 2a). As an example, the difference in reproducibility values between replicates of K for PE and IC preparation was 11.9%; for W5 it was 41.9%, and for W11 it was 3.7%. The variety of these isolates (W5 is a *L. pneumophila* strain unrelated to K, and W11 belongs to a separate species) most likely had an effect on the relative effectiveness of the two preparations to properly isolate and/or concentrate cellular components for analysis. It was noted during PE preparations that pelleted cells from certain environmental isolates showed more resistance to dissolving after application of formic acid and acetonitrile than others, including W5 and W11. This could be indicative of phenotypic variation, such as membrane composition, amongst *Legionella* environmental strains having an effect on sample preparation quality depending on the preparation method used, and thus, any MALDI-TOF-MS analysis based on data from these samples. In addition to this, environmental isolates of *Legionella* can have greatly different growth kinetics from well-established lab strains represented in varying nutritional requirements, increased incubation time, and reduced culturability [27]. This variation on growth rates was observed amongst several environmental isolates used in this study, e.g., W11 produced mature colonies within 48 h of culturing, whereas W12 took up to 120 h. While the importance of organism-dependent MALDI-TOF-MS methods selection has been established [28], results from this study further highlight this significance of methods optimization for the characterization of not only species, but also strains of *Legionella* and, potentially, other microbes.

The majority of studies on *Legionella* analysis via MALDI-TOF-MS have utilized smear preparations [4,13,14,15], a method which sacrifices sample quality for lower preparation time and cost. A study to determine optimal protein extraction preparation methods for inactivating pathogenic bacteria [29] determined that the use of ethanol and formic acid versus other solvents resulted in a greater number of higher intensity peaks for *Legionella* samples. In another study focused on typing environmental *Legionella* [16] two protein extraction methods were employed: (1) a vortex method involving the use of TFA and a 0.2 µm filter to isolate proteins, and (2) a method involving the use of a bead beater on cell suspensions frozen in liquid nitrogen, with the former proving superior for use in MALDI-TOF-MS analysis. The improved quality of spectra generated by the use of optimized methods for *Legionella* isolates, particularly protein extraction preparation, from our own and others’ studies demonstrates the importance of utilizing ideal methodology for high-resolution characterization of this organism via MALDI-TOF-MS.

### 3.2. MALDI-TOF-MS Cluster Analysis

**Figure 1:**
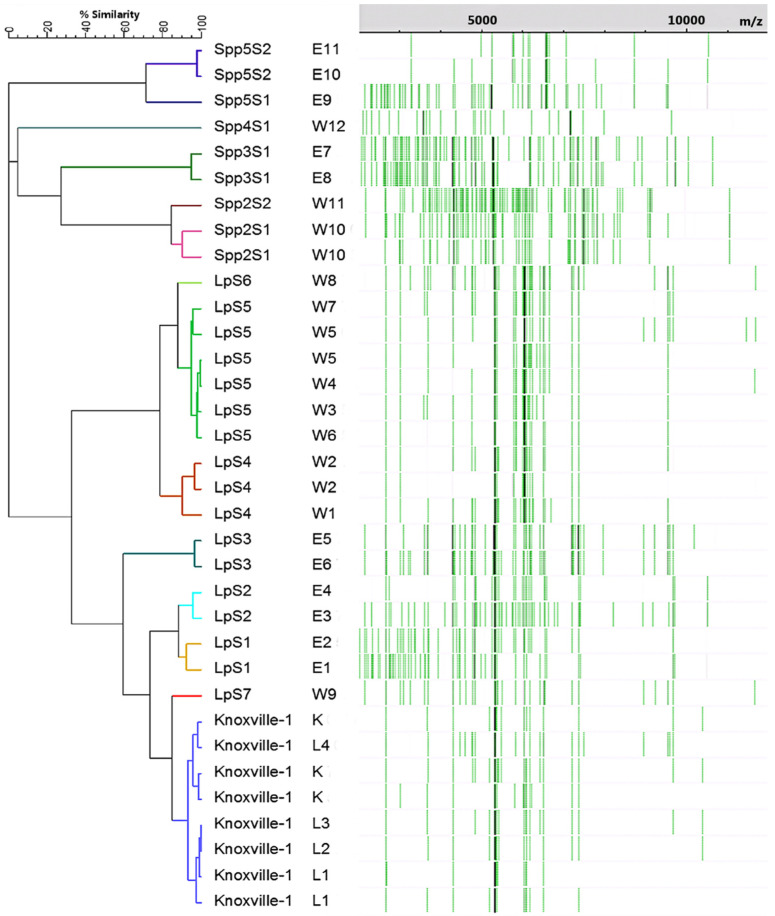
Dendrogram created via cluster analysis of summarized mass spectra from three technical replicates of 34 cultures of 28 *Legionella* isolates, along with respective mass spectra band data. Similarity coefficients were calculated using peak-based Pearson correlations, and clustering was performed via complete linkage clustering. Cultures K and L1–L4 are isolates of the lab strain Knoxville-1. Samples E1–11 and W1–W12 are environmental isolates from east and west central AZ, respectively. Environmental isolates are labeled as members of *L. pneumophila* (Lp) or other *Legionella* species (Spp.) followed by presumptive strain group numbers (e.g., S1) based on the analysis. Terminal branches are color-coded based on presumptive strain groups.

**Figure 2:**
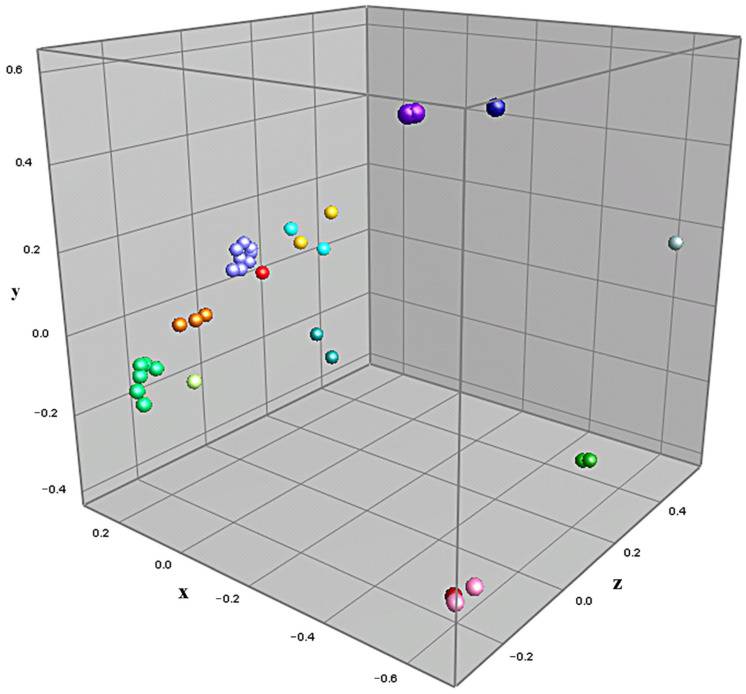
MDS scatterplot, with optimized positioning, based on cluster analysis described in Figure 1. Data points are color-coded based on presumptive strain groups.

**Figure 3:**
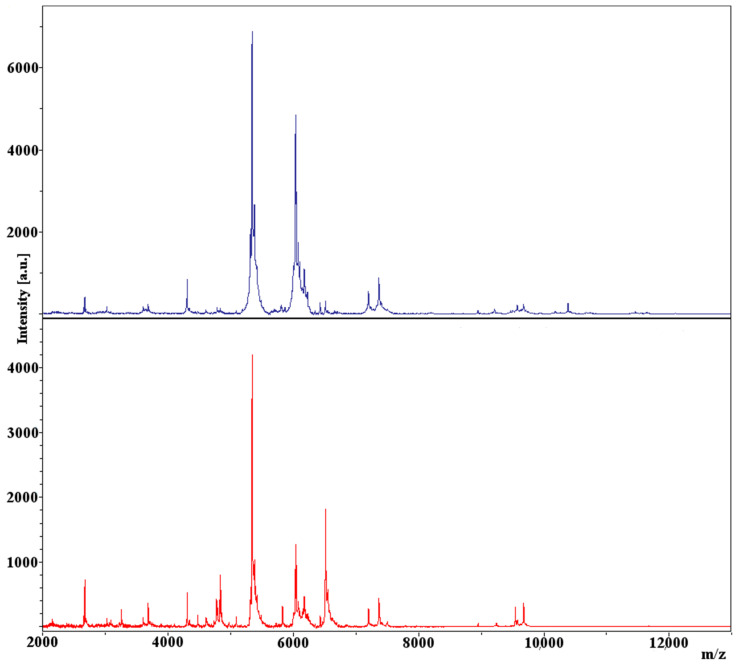
Representative spectra generated from samples of isolates of Knoxville-1 (**top**) and W9 (**bottom**).

Cluster analysis was performed on samples of *Legionella* isolates prepared via methods previously determined to be optimum (PE preparation from agar cultures), with the goal of achieving strain-level characterization. Figure 1 displays the dendrogram constructed via cluster analysis of the 28 analyzed isolates, along with each culture’s respective summary spectra, represented in a pseudo-gel view. Branches for each culture are color-coded based on presumptive strains predicted using a 90% similarity cut-off. Figure 2 displays the results of the cluster analysis in the form of a multidimensional scaling (MDS) scatterplot, carrying over the color-coding assigned in the dendrogram of Figure 1. Statistical inference was performed on the presumptive strains assigned during cluster analysis via jackknifing, with the goal of determining rates of correct classification for assigned strains. Representations of two of the spectra used to generate Figure 1 and Figure 2 are shown in Figure 3.

Using the species and strain cut-off limit of 32 and 90% similarity, respectively, the cluster analysis revealed 4 non-*pneumophila* species and 14 strains of *Legionella* amongst the 28 isolates examined. All 8 Knoxville-1 cultures (including the stock culture) clustered closely and distinctly with 5 isolates, with an average similarity of approximately 93%. Replicate cultures of the 6 *L. pneumophila* East isolates formed three strains, while the 9 *L. pneumophila* West isolates formed 4 strains. The 5 East and 3 West non-*pneumophila* isolates formed 4 separate species, two of which had two distinct strains amongst their isolates. *L. pneumophila* environmental isolates (with the exception of W9, which grouped close to Knoxville-1 isolates) formed distinct clusters based on the sampling site. Five West *L. pneumophila* isolates formed a single strain, with an average similarity of 94.6%. No replicate isolate cultures clustered apart and no *L. pneumophila,* and non-*pneumophila* isolates clustered together. Figure 2 depicts definitive, near equidistant, separation of isolates by species, along with clustering of strains and species in accordance assigned via cluster analysis similarity, with the possible exception of isolates belonging to LpS1 and LpS2 (isolates E1–E4), which appear to be evenly spaced amongst themselves. Jackknife analysis resulted in 100% rates of correct classification for all presumptive strains, with the exception of LpS1 and LpS2, which were incorrectly classified as each other at a rate of 50%.

The clearly defined clustering of isolates depicted in Figure 1 and Figure 2, including those of *L. pneumophila* by sampling site, suggest that the cluster analysis performed on MALDI-TOF-MS spectra of environmental *Legionella* isolates was able to characterize these isolates to the strain level. These results are validated by several factors. The high similarity values amongst multiple Knoxville-1 cultures and isolates, as well as replicate cultures of environmental *Legionella*, demonstrate an appropriate level of accuracy and precision in sample preparation, data acquisition, and data analysis methods performed in the study. The lack of incorrect clustering between known *pneumophila* and non-*pneumophila* isolates suggests a high level of accuracy for the method in differentiation of these organisms at the species level. The agreement in clustering between Figure 1 and Figure 2, as well as results from the jackknife analysis (with the notable exception of strains LpS1 and LpS2), show a reasonable level of confidence in the predicted strains. This is demonstrated particularly well for isolates of strains Knoxville-1 and LpS5, both of which clustered tightly and distinctly amongst themselves, despite the presence of other relatively similar strains (W9 and W8, respectively).

This study has yielded the following novel results: strain level profiling of *Legionella* isolated from tap water and belonging to multiple species, typing of the greatest number of *Legionella* strains in a single study, and sampling site-dependent typing of several strains of *Legionella*. Differentiation of the 5 *Legionella* species was reliably achieved, regardless of clustering approaches (data not shown) or preparation and culturing methods, which were optimized for the strain typing results generated. This helps to explain why species level characterization has been well documented for *Legionella*, including environmental isolates, [4,13], but strain-level profiling of these bacteria has only been rarely reported [3,16,17]. The level of similarity and tight clustering between isolates W3–7 not only suggests a high level of resolution for the analysis used, but also demonstrates the potential for applied typing of environmental *Legionella*. Isolates W3, W4, W6, and W7 were cultured from automobile washer fluid in four separate vehicles prepared using tap water originating from the same faucet that produced the tap water isolate W5 was cultured from. Even more significant is the fact that three additional isolates from separate vehicles, W1, W8, and W9, along with W2, a second isolate from the same tap water as W5, all belonged to strains distinct from W3–W7. The fact that multiple strains of *L. pneumophila* originating from the same source were able to be distinguished suggests that MALDI-TOF-MS could be used in a variety of applications, such as tracking the source of contamination of a *Legionella* strain responsible for an outbreak of Legionnaires’ Disease.

The fact that isolate W9 clustered so closely to isolates of Knoxville-1 is interesting. In addition to spectra appearing qualitatively different between the isolates (Figure 3), in depth analysis of spectra from isolate W9 (data not shown) revealed it to be definitively distinct from K, possessing peaks not seen in K (i.e., at 9546 Da), while lacking peaks found in Knoxville-1 isolates (i.e., at 10,386 Da). These results would seem to indicate that W9 is an environmental strain of *L. pneumophila* incidentally more closely related to the type strain Knoxville-1 than to the other environmental strains isolated from the same source in this study. Several strain and species designations that were assigned in this study were not as clearly definitive as others, including the strain separations of LpS1/LpS2, LpS5/LpS6, and Spp2S1/Spp2S1, as well as the species separation of Spp2/Spp3. While all of these separations were assigned due to their respective isolates falling below the cluster analysis similarity cut-off values determined from known strains and species, the isolates in question were all within 5% of the requisite similarity for same species/strain designation (1.2% for LpS1/LpS2). The three-dimensional view of the relationships between isolates in Figure 2 suggest that distinct clustering and, thus, potential strain/species distinction, did occur for these isolates, with the possible exception of LpS1/LpS2. The four isolates belonging to these assigned strains appear to be relatively evenly spaced amongst themselves, suggesting they may form a single distinct strain, albeit with isolates relatively less related when compared to those of the other presumptive strains of the study. This idea is further supported by the 50% rate of correct classification between LpS1/determined via jackknife analysis. The potential single strain status of these four isolates indicates that they may be an example of a group of *Legionella* near the limit of detection for strain level profiling using the methods performed, although higher resolution spectra data generated through improved methodology may result in this distinct strain separation amongst them.

### 3.3. 16S rRNA Analysis of Isolates

Analysis of the 16S rRNA sequences from selected isolates agreed with the putatively assigned strain and species designations determined via MALDI-TOF-MS spectra analysis, with the exception of E5. All other predicted strains contained one or more base pair differences between all other isolates examined. As an example, the 16S rRNA sequence of strain K differed from strain W5, but the sequences of W5, W6, and W7 were identical. The greatest differences occurred between the two non-*pneumophila* species and all other isolates examined, while the fewest were observed between W8 and W5/W6/W7. Strain K displayed differences between all other isolates examined, indicating a lack of environmental sample contamination due to cross-contamination. Two base pair differences were present between strains E2 and E4, supporting their cluster analysis-based designation as separate strains. Isolate E5 produced a sequence identical to three west sampling site isolates belonging to one putative strain, W5, W6, and W7. Due to the relatively small amplicon of the sequence and conserved nature of this gene, combined with MADLI-TOF-MS spectra being distinct between these two putative strains, it is predicted that the similarities in the 16S rRNA gene region examined were coincidental.

## 4. Conclusions

Through the systematic optimization of sample preparation methodology, strain level profiling of both *L. pneumophila* and non-*pneumophila Legionella* species was achieved. Results from this study have implications for a number of fields including public health, environmental engineering, and microbial ecology. Applications would not only extend to *Legionella*, but other environmental microbes as well: the ability to reliably type microbes isolated from various environmental sources in a cost-efficient and timely manner would be greatly beneficial to those interested in tracking disease progression, monitoring drinking water, or studying microbial population dynamics. As resolution of MALDI-TOF-MS profiling of microbes continues to improve, the use of this technology will undoubtedly be investigated for further commercial and academic applications, warranting further research into improving its use in typing beyond the species level. For *Legionella* in particular, further improvement of methodology, analysis of an increased number of environment and type-strain isolates, and comparison to established typing methods will help to further research in this area.

## Figures and Tables

**Table 1:** Isolates of *Legionella* used in the study ^a^.

Isolate	K	L1–L4	E1–E4	E5, E6	E7–E11	W1, W5	W2–W4, W6–W9	W10, W11	W12
Water Source	Stock Culture	Laboratory Experiment	East System	East System	East System	West System	West System	West System	West System
Water Type	N/A	Sterile Water	Tap Water	Tap Water	Tap Water	Tap Water	Washer Fluid	Washer Fluid	Tap Water
Spp	Lp	Lp	Lp	Lp	Non-Lp	Lp	Lp	Non-Lp	Non-Lp

^a^ Isolate species were determined via PCR. Isolates K and L1–L4 were laboratory strain *L. pneumophila* Knoxville-1 ATCC strain 33153, while all others were environmental isolates. Lp refers to *L. pneumophila* and Non-Lp indicates *Legionella* species other than *L. pneumophila*.

**Table 2:** (**a**) Comparisons of spectra quality parameters in IC vs. PE preparations with agar culturing for three isolates of *Legionella* ^a^. (**b**) Spectra quality parameters in PE preparations with broth culturing for three isolates of *Legionella* ^b^.

Sample	Base Peak S:N Ratio	Base Peak Resolution	Peak Number	Reproducibility (%)
(**a**)				
K IC	364.4 ± 275.2	627.0 ± 30.2	94.7 ± 156.2	80.9 ± 6.5
K PE	480.9 ± 219.0	647.6 ± 35.0	23 ± 2.6	92.8 ± 4.7
W5 IC	616.9 ± 657.7	650.2 ± 31.1	120.0 ± 79.2	83.5 ± 9.6
W5 PE	327.1 ± 188.9	655.3 ± 27.5	33.3 ± 0.6	87.2 ± 8.8
W11 IC	103.0 ± 44.4	742.1 ± 47.7	444.3 ± 275.2	50.0 ± 23.7
W11 PE	275.4 ± 24.9	782.8 ± 68.8	104.0 ± 32.9	91.9 ± 1.2
Avg IC	361.4 ± 256.9	673.1 ± 60.9	219.7 ± 194.9	71.5 ± 18.6
Avg PE	361.1 ± 106.9	695.2 ± 75.9	53.4 ± 44.1	90.7 ± 2.9
(**b**)				
K Broth	140.3 ± 28.8	521.2 ± 9.7	765.3 ± 71.9	76.6 ± 9.1
W5 Broth	117.6 ± 46.3	546.3 ± 49.7	772.7 ± 42.2	45.7 ± 14.7
W11 Broth	40.4 ± N/A	707.6 ± N/A	1193 ± N/A	N/A
Avg Broth	99.4 ± 52.4	591.7 ± 101.1	910.3 ± 244.8	61.2 ± 21.9

^a^ Data shown represent averages of triplicate samples from a single preparation followed by their standard deviations. Results for all parameters show a significant difference between IC and PE with the exception of W5 base peak resolution and reproducibility. ^b^ Data shown represent averages of triplicate samples from a single preparation followed by their standard deviations. Standard deviations and reproducibility are not available for W11 due to only one of three samples producing an analyzable spectrum.

## Data Availability

Data from this study are available from the corresponding author upon reasonable request.
